# Measuring value in healthcare from a patients’ perspective

**DOI:** 10.1186/s41687-021-00364-4

**Published:** 2021-10-12

**Authors:** Stafford Dean, Fatima Al Sayah, Jeffrey A. Johnson

**Affiliations:** 1 Alberta Health Services, Edmonton, AB Canada; 2 Alberta PROMs and EQ-5D Research and Support Unit (APERSU), School of Public Health, University of Alberta, Edmonton, AB Canada

## Introduction

Throughout the world there is a growing recognition that the patient’s perspective is highly relevant to efforts to deliver high-value patient-centered care and to improve the quality and effectiveness of healthcare. One of the key challenges to achieving this is the limited measurement of outcomes that matter most to patients. At an OECD conference in 2017, Ministers of Health from around the world stated, “We need to invest in measures that will help us assess whether our health systems deliver what matters most to people” [1]. The introduction of patient-reported outcome measures (PROMs)—measurement instruments designed to assess the status of a patient’s health condition that comes directly from the patient [2]—is one strategy to ensure that patient’s perspectives are systematically incorporated into the approaches of delivering healthcare services, and valuing the performance of the healthcare system [3–6]. PROMs could act to improve the quality of care in the same way as any other benchmarking tool [7], and some suggest that PROMs have the potential to transform healthcare [3].

There has been an increased use of PROMs in health systems around the world, a movement that started and has been led by the National Health System (NHS) in the UK [7]. In Canada, the Canadian Institute for Health Information (CIHI) and the *Alberta PROMs and EQ-5D Research and Support Unit* (APERSU) are leading similar efforts to establish national and provincial PROMs strategies, and support the use of PROMs in the Canadian healthcare system [8, 9].

## The Alberta context

The province of Alberta is home to nearly 4.4 million Canadians, and, like Canada’s other provinces and territories, has a publicly funded healthcare system. Alberta Health Services (AHS) is the organization through which the provincial health ministry (Alberta Health) provides health care to Alberta residents. AHS is Canada’s first and largest province-wide fully integrated health system, is the provincial organization responsible for delivery of hospital, long-term care and community health care services to Albertans. AHS is the largest employer in Alberta with over 103,000 direct employees and thousands of other affiliated healthcare personnel and volunteers, with an annual operating budget for 2019–2020 fiscal year of $15.4 billion CAD [10].

AHS monitors the performance of the health system using 13 performance measures grouped into four categories: (1) improving patients and families experiences; (2) improving patient and population health outcomes; (3) improving the experience and safety of AHS employees; and (4) improving financial health and value for money [11]. AHS, like many other healthcare systems, collects large amounts of healthcare data to inform various performance measures; however, what is often lacking is understanding how the system is performing from a patients’ perspective. In order to meaningfully assess value, measuring outcomes—especially those that matter to patients—is imperative. As such, PROMs have been increasingly playing a crucial role in outcome measurement within the Alberta healthcare system.

AHS has embarked on a data acquisition strategy across the domains of the triple aim framework: improving the health of populations, improving the patient experience, and reducing per capita cost of care [12]. PROMs add a critical dimension by measuring outcomes from the patient’s perspective, which allow us to assess what we produce in terms of health impact based on what matters most to patients, not just healthcare utilization. Additionally, PROMs align with key foundational strategies for AHS, especially the patient and family first strategy that strives to ensure practices place families and patients at the centre of all activities and improves their health and wellness [13]. Hearing directly from patients on outcomes and experiences of health system encounters is key to that strategy.

AHS has supported the use of PROMs within the healthcare system first by selecting and endorsing the EQ-5D (Additional file 1) as the generic PROM for use in Alberta in 2015, and embedding the ability to capture the EQ-5D and other PROMs in *Connect Care*, the province-wide electronic patient medical information system. Among commonly available generic PROMs, the EQ-5D was selected by AHS for 4 main reasons: (1) evidence of adequate measurement properties in most applications (also recognizing expected improvements with the new 5-level version); (2) Canadian preference-based scoring function for application in economic/value-based evaluations; (3) brevity; 4: local expertise to provide support to end-users in the province. To this fourth point, AHS partnered with the EuroQol Research Foundation, the developer of EQ-5D instruments, and supported the establishment of a centre of excellence at the University of Alberta called *Alberta PROMs and EQ-5D Research and Support Unit* (APERSU) to support and enhance the use of PROMs in Alberta’s healthcare system, and conduct research to inform local implementation of PROMs programs and strategies.

The standardization of generic PROM measurement in Alberta, enabling systematic measurement through patient’s electronic medical record, developing provider buy-in and resource investment, and the establishment of a local center of excellence supporting PROMs end-users have accelerated and advanced the use of PROMs in various clinical settings in our province, which will allow sharing data on outcome more efficiently as well as comparisons that will accelerate care improvement. Currently, the EQ-5D, along with disease-specific PROMs in some clinical areas are being widely collected across the healthcare system. PROMs implementation varies across clinical settings, with varying levels of integration into clinical workflows, and data use at the micro, meso and macro levels within the system [14].

## PROMs and value-based care

Health systems tend to be held to account for outcomes that are administrative in nature, such as balancing budgets, improving wait time improvements, service volume targets, or adding health care capacity. The use of PROMs enables the system to move closer to managing outcomes as opposed to administrative activities. There is a lot of focus on cost in Alberta’s health system given the fiscal challenges. Alberta went from being one of the lowest spending provinces on health care in the 1990’s to one of the highest, with an average health spending of $7658 CAD per person in 2019, and projections of continued increase over the coming years [15]. We need to examine costs together with outcomes, as a cost without an outcome is interesting at best. The movement to capturing outcomes and cost will lead to much better judgements on where to reduce expenditures. PROMs provide a valuable source of evidence to complement and enhance outcome measurement frameworks that will help establish whether the Alberta healthcare system services are effective and a good value-for-money from patients’ perspectives.

Funding systems may also be tied to outcomes. Alberta funding mechanisms are largely based on global budgets and Fee For Service funding systems [16]. As we develop PROMs there is an opportunity to enhance and incentivise funding systems that promote outcomes. For instance, PROMs data being collected in the system could be used in evaluating, monitoring, and improving provider performance, setting performance standards and benchmarks, and identifying the poorest and best performing providers. This in turn would inform funding systems, and enhance value-based care. Ultimately the health care system goal to enable longer and better quality of lives and that goal can be better gauged with the formal use PROMs.

## Final remarks

Despite successful PROMs implementation by many users within the Alberta health system and significant investment from the provincial healthcare system, several operational and methodological challenges continue to exist. Investigating the best approaches of collecting PROMs data including timing and frequency of data collection in various clinical areas, exploring effective ways of interpreting and reporting PROMs data to clinicians and patients, using PROMs data to support performance evaluation and value of care, and identifying the most appropriate risk-adjustment methods, as well as exploring methods for using PROMs to support decision-making around healthcare planning and resource allocation are areas that require further investigation and development. We continue our efforts alongside leading PROMs researchers and stakeholders in our province alongside national and international collaborators to address these questions and support initiatives to generate evidence to further enhance the use of PROMs within healthcare systems in Canada and internationally.

In this supplement, we share eight papers starting with one introducing a multi-level approach that we have adopted for the use of PROMs data within the healthcare system in Alberta, followed by a paper on a strategy for the selection of PROMs for their use in health systems, and then a series of papers reporting on the implementation of PROMs in various clinical settings within the healthcare system in Alberta. This supplement is complemented by a closing commentary by PROMs leaders at the Canadian Institute of Health Information (CIHI) highlighting national PROMs initiatives, and a look into the future for using PROMs in Canada.

## Supplementary Information

**Additional file 1**. Appendix: EQ-5D-5L Instrument.

## Data Availability

Not applicable.

## Abbreviations

OECD: Organization for Economic Cooperation and Development
PROMs: Patient-reported outcome measures
AHS: Alberta health services
APERSU: Alberta PROMs and EQ-5D Research and Support Unit
CIHI: Canadian Institute of Health Information
