# Supplementary material for: Measuring value in healthcare from a patients’ perspective
Source: J Patient Rep Outcomes. 2021 Oct 12;5(Suppl 2):88. doi: 10.1186/s41687-021-00364-4 (PMC8511223; doi:10.1186/s41687-021-00364-4)
Supplement: Supplementary file 1 — Additional file 1. Appendix: EQ-5D-5L Instrument. [file 41687_2021_364_MOESM1_ESM.pdf]

EQ-5D-5L

Health Questionnaire

English version for Canada

Canada (English) v.2 © 2009 EuroQol Group. EQ-5D™ is a trade mark of the EuroQol Group

Under each heading, please tick the ONE box that best describes your health TODAY

MOBILITY

I have no problems in walking ❑

I have slight problems in walking ❑

I have moderate problems in walking ❑

I have severe problems in walking ❑

I am unable to walk ❑

SELF-CARE

I have no problems washing or dressing myself ❑

I have slight problems washing or dressing myself ❑

I have moderate problems washing or dressing myself ❑

I have severe problems washing or dressing myself ❑

I am unable to wash or dress myself ❑

USUAL ACTIVITIES *(e.g. work, study, housework, family or leisure activities)*

I have no problems doing my usual activities ❑

I have slight problems doing my usual activities ❑

I have moderate problems doing my usual activities ❑

I have severe problems doing my usual activities ❑

I am unable to do my usual activities ❑

PAIN / DISCOMFORT

I have no pain or discomfort ❑

I have slight pain or discomfort ❑

I have moderate pain or discomfort ❑

I have severe pain or discomfort ❑

I have extreme pain or discomfort ❑

ANXIETY / DEPRESSION

I am not anxious or depressed ❑

I am slightly anxious or depressed ❑

I am moderately anxious or depressed ❑

I am severely anxious or depressed ❑

I am extremely anxious or depressed ❑

Canada (English) v.2 © 2009 EuroQol Group. EQ-5D™ is a trade mark of the EuroQol Group

- We would like to know how good or bad your health is TODAY.
- This scale is numbered from 0 to 100.
- 100 means the best health you can imagine.
  0 means the worst health you can imagine.
- Mark an X on the scale to indicate how your health is TODAY.
- Now, please write the number you marked on the scale in the box below.

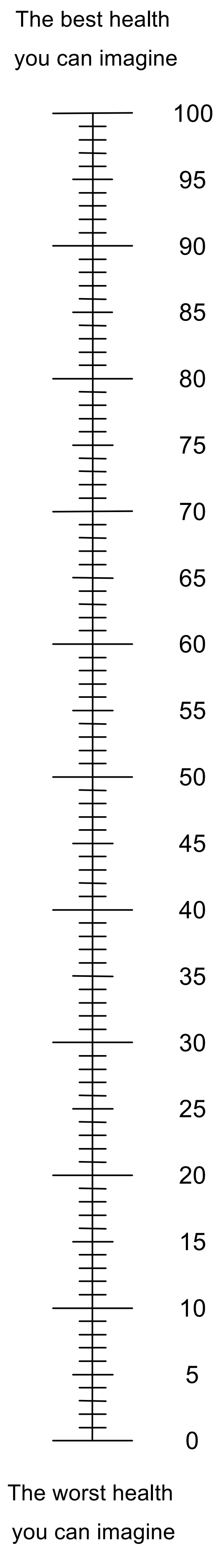

YOUR HEALTH TODAY =

Canada (English) v.2 © 2009 EuroQol Group. EQ-5D™ is a trade mark of the EuroQol Group
